# Pilot study: assessing the clinical diagnosis of allergy in atopic children using a microarray assay in addition to skin prick testing and serum specific IgE

**DOI:** 10.1186/s12948-016-0046-z

**Published:** 2016-08-19

**Authors:** Ru-Xin Foong, Graham Roberts, Adam Tobias Fox, George du Toit

**Affiliations:** 1 Division of Asthma, Allergy and Lung Biology, Guy’s and St. Thomas’ NHS Foundation Trust, London, SE1 7EH UK; 2 Institute of Child Health, University College of London, London, UK; 3 University of Southampton and National Institute for Health Research Respiratory Biomedical Research Unit, Southampton and David Hide Centre, Southampton, UK

**Keywords:** Aeroallergens, Atopic dermatitis, Diagnostic investigations, Food allergy

## Abstract

**Background:**

Children with atopic dermatitis (AD) are at risk of developing allergy. Alongside clinical history, testing modalities include skin prick tests (SPT), specific immunoglobulin-E (sp-IgE) and recently, microarray assays. The aim of this pilot study was to assess current tests and the ISAC sIgE-112 system in the diagnosis of food and aeroallergen allergy.

**Methods:**

Children aged 0–11 years with moderate to severe AD were included. An initial allergy assessment including clinical history, SPT and sp-IgE was performed to determine food and aeroallergen sensitization. A second independent clinical assessment using the same information given to the first assessor and ISAC test results for food and aeroallergen sensitization was also made for each participant. The results from both were compared.

**Results:**

30 children [mean age 3.91 years (SD 3.3)] were included; 53.3 and 46.7 % had moderate and severe AD, respectively. Sp-IgE tests had a higher percentage of positive results compared to SPT and ISAC tests for common allergens. There was a significant difference between the three tests in detecting aeroallergen sensitization (p = 0.038), especially between sp-IgE and ISAC tests, but no significant difference between the tests for food allergen sensitization. There was good agreement between the two assessors; 70 % of the children had a change in diagnosis, with 60 % having at least one diagnosis added and 40 % having at least one diagnosis removed.

**Conclusions:**

There is a role for the use of ISAC testing in diagnosing sensitization and allergy in children with AD as it leads to a change in diagnosis for many patients. Further work is required to assess its clinical and cost effectiveness.

**Electronic supplementary material:**

The online version of this article (doi:10.1186/s12948-016-0046-z) contains supplementary material, which is available to authorized users.

## Background

Atopic dermatitis (AD) is a common inflammatory skin disease affecting 15–20 % of children in developed countries [1]. It usually starts in the first few months of life and is frequently outgrown by 15 years of age although there is a large amount of variability between children [2].

It is well known that there are close links between food allergy and AD as it is a significant risk factor for the development of food sensitization and food allergy [2–4]. Patients with higher levels of total Immunoglobulin-E (IgE) in their blood, which is often the case in children with AD, are more likely to be sensitised to foods [1, 5]. Kumar et al. found that children with AD had an 8.4-fold higher risk of developing a food allergy [6]. de Benedictis et al. demonstrated that of infants with AD in various countries around the world, 55.5 % were sensitised to at least one allergen and 52.9 % had a raised serum total IgE [7].

Due to the increased likelihood of children with AD being sensitised to various food allergens, it can be difficult to establish whether these children have true food allergy. The European Academy of Allergy and Clinical Immunology (EAACI) guidelines recommend the use of clinical history, skin prick tests (SPT) and serum specific IgE (sp-IgE) tests in the investigation of food allergy but the gold standard for diagnosis remains the oral food challenge [8]. AD is often associated with aeroallergen sensitization and in the context of a high level of total IgE, there are often low levels of sp-IgE to numerous aeroallergens [1]. These are of unclear clinical significance.

Immunocap^®^ ISAC-112 (Thermo Fisher/Phadia AB, Uppsala, Sweden) is an in vitro assay for the measurement of allergen sp-IgE antibodies in human plasma [9]. It is intended to aid in the diagnosis of IgE-mediated allergic disorders. There is currently no advice on the use of ISAC testing worldwide [8, 10]. The National Institute of Allergy and Infectious Diseases (NIAID) guidelines do not recommend the combined use of SPTs and sp-IgE tests for routine diagnosis of food allergy and instead recommend individual SPT and sp-IgE tests to help identify foods that might be provoking a reaction alongside clinical history [10]. Thus, the utilization of a broad screening test is controversial. Whilst it may facilitate a more accurate clinical allergy diagnosis for specific atopic conditions [11, 12], it has been suggested that identifying patterns of allergen responses for patients with AD is less reliable [12]. Spergel et al. [13] found that the predictive values of food-antigen sp-IgE were not clinically significant in predicting the development of food allergy in children with mild to moderate AD. However, this study performed sp-IgE only for common food allergens, potentially missing less common allergens [13].

The ISAC-112 has the potential as a single test modality, to identify food and aeroallergen sensitizations in at risk atopic children. Hatzler et al. [14] looked at the IgE response to grass pollen antigens and demonstrated that sensitization can occur years before the onset of clinical disease through a process called “molecular spreading”. Therefore, the use of microarrays could be convenient in the setting where common food allergens have not yet been introduced thereby limiting the value of a detailed clinical history.

The aim of this pilot study was to assess the use of current diagnostic modalities (clinical consultation, SPT, sp-IgE) in a specialist allergy clinic in children with AD. We also assessed whether Immunocap^®^ ISAC sIgE-112 testing could improve the diagnosis of food and aeroallergen allergy in these children.

## Methods

### Participants

We recruited children aged 0–11 years from the paediatric allergy clinic at St. Thomas’ Hospital, London UK, an urban, tertiary referral centre. We identified children who had moderate to severe AD using any one of the LEAP study criteria [15]: parents or guardians’ description of their child’s AD severity as moderate or severe; use of topical creams and ointments containing corticosteroids or calcineurin inhibitors (defined as severe); or modified Scoring Atopic Dermatitis System (SCORAD) evaluation of at least 15 [16]. Written consent was obtained from the parents and child (if age relevant). Ethics approval was given by the NRES Committee London—Fulham (12/LO/1733).

### Initial allergy assessment

The participants who fitted the criteria for moderate to severe AD underwent a detailed allergy clinical assessment (clinical history, SPT, sp-IgE tests) by a paediatric allergy consultant. A clinical diagnosis of food allergy and clinically significant aeroallergen sensitization was made based on these findings. The most common food and aeroallergens were tested for: peanut (Ara h1, Ara h2, Ara h3, Ara h8, Ara h9), egg (white or yolk), cow’s milk, timothy grass pollen, birch tree pollen, *Dermatophagoides pteronyssinus* house dust mite, *Alternaria* mould, cat and dog. SPT were performed using Alyostal^®^ Stallergenes allergen extracts by trained paediatric allergy nurses in the department. Sensitization to food and aeroallergens to the same allergens was measured by specific-IgE (Thermo Fisher, Uppsala, Sweden). Food allergy was defined by a history of IgE-mediated symptoms (e.g. rash, urticaria, wheeze, vomiting, hypotension, anaphylaxis) on exposure to a food antigen, in the presence of a positive SPT (≥3 mm) or sp-IgE (≥0.35 kU/l) [17]. Clinically significant aeroallergen sensitization was defined as positive SPT (≥3 mm) or sp-IgE (∓0.35 kU/l) in the presence of a history of worsening AD, allergic rhino-conjunctivitis or asthma symptoms when exposed to the relevant aeroallergen.

### Second allergy assessment with ISAC

All participants then had an ISAC-112 (Thermo Fisher Scientific©) test performed of which blood was taken after the clinic appointment. Immunocap and ISAC-112 analysis were processed by the Viapath Analytics department at King’s College Hospital, London UK. A positive ISAC was defined as ≥0.3 ISU-E for the same allergens tested for in the initial assessment. A second assessment was performed by a second paediatric allergy consultant who used the clinical history, SPT, sp-IgE and ISAC results to make an independent clinical assessment of the presence of food allergy and aeroallergen sensitizations.

### Statistics

Statistical analysis was performed using (STATA, version 14, College Station, USA). Descriptive statistics were presented as means with standard deviation (SD) where appropriate and categorical variables presented as percentages. Kappa statistics were used to look at agreement between the tests results and allergic diagnoses. A Kappa of 0.01–0.4 is considered slight to fair agreement, 0.41–0.60 is moderate agreement and 0.61–0.99 is substantial to almost perfect agreement [18]. Chi square tests were used to compare the proportion sensitised to food allergens and aeroallergens based on the test performed. To compare the number of allergens that were positive for each participant for the difference tests and number of allergic diagnoses per participant, repeat measures ANOVA tests were used.

## Results

A total of 30 participants were recruited for this study. The mean age of the children was 3.91 years (SD 3.3) and 60 % (n = 18) were male. Of the group, 53.3 % (n = 16) and 46.7 % (n = 14) had SCORAD scores suggestive of moderate and severe AD, respectively.

### Comparison of SPT, sp-IgE and ISAC tests

**Fig. 1:**
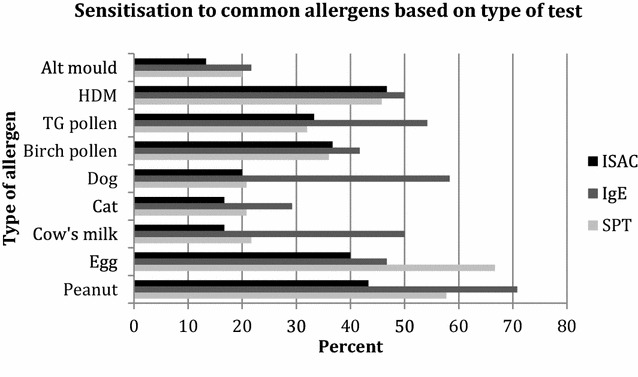
Sensitization to common allergens based on the type of test performed. Figures are numerator/denominator (%) positive for each test for allergic sensitisation. Positive SPT was defined as ≥3 mm, positive specific IgE as ≥0.35 kU/l and positive ISAC as ≥0.3 ISU-E

We compared the percentage of children who had a positive test result on SPT, sp-IgE and ISAC for common food and aeroallergens (Fig. 1). Overall, apart from egg allergen, the sp-IgE tests had a higher percentage of positive results for each allergen compared to SPT and ISAC tests. When comparing SPT and ISAC tests a relatively similar percentage of positive results for allergens were noted. For example, for birch pollen, 36 % had a positive SPT and 36.7 % had a positive ISAC test.

We also looked at the agreement between the three different tests (Table 1). There was good agreement between the SPT and ISAC tests for sensitization to selected food and aeroallergens. There was relatively good agreement between sp-IgE and ISAC tests for house dust mite and *Alternaria* mould. There was also good agreement between SPT and sp-IgE for house dust mite. Other test and allergen comparisons showed low levels of agreement.

**Table 1:** Agreement between the tests based on sensitization

Allergen	SPT and IgE	SPT and ISAC	IgE and ISAC
Peanut	0.42 (0.02)	0.54 (0.003)	0.36 (0.023)
Egg	0.29 (0.072)	0.43 (0.01)	0.19 (0.148)
Cow’s milk	0.39 (0.010)	1.00 (<0.001)	0.38 (0.006)
Cat	0.53 (0.007)	0.86 (<0.001)	0.56 (0.003)
Dog	0.30 (0.029)	0.18 (0.192)	0.38 (0.008)
Birch pollen	0.20 (0.175)	0.65 (0.001)	0.55 (0.003)
Timothy grass pollen	0.28 (0.063)	0.73 (<0.001)	0.44 (0.005)
House dust mite	0.73 (<0.001)	0.58 (0.002)	0.92 (<0.001)
*Alternaria* mould	−0.25 (0.855)	0.06 (0.390)	0.70 (<0.001)

Figures are Kappa statistics (p values) where represent the level of agreement between allergen sensitization tests having taken into account any agreement that is expected by chance. Results of 0.61–0.99 signify excellent agreement, 0.41–0.60 signifies moderate agreement and 0.01–0.4 signifies only slight to fair agreement [18]

### Comparison of positive sensitizations for each participant

The number of positive sensitizations for each participant for each test was compared. For aeroallergens, overall there was a significant difference between the results of the three tests (p = 0.038); subsequent pairwise comparisons found that the only significant difference was between the sp-IgE and ISAC tests results (Table 2). For food allergens, there was no overall significant difference found between the results from the different tests (p = 0.469). These results do require further consideration in the context of the specificity of the tests.

**Table 2:** Number of food and aeroallergen sensitizations by participant based on the type of test performed

Number of sensitizations	SPT (%)	Specific IgE (%)	ISAC (%)	P value
*Aeroallergens*				
0	13 (43.3)	9 (30.0)	8 (26.7)	0.038*
1	7 (23.3)	3 (10.0)	10 (33.3)	
2	3 (10.0)	6 (20.0)	1 (3.3)	
3	2 (6.7)	7 (23.3)	6 (20.0)	
4	2 (6.7)	1 (3.3)	5 (16.7)	
5	3 (10.0)	2 (6.7)	0 (0)	
6	0 (0)	2 (6.7)	0 (0)	
*Food allergens*				
0	7 (23.3)	8 (26.7)	11 (36.7)	0.469
1	13 (43.3)	8 (26.7)	11 (36.7)	
2	7 (23.3)	6 (20.0)	5 (16.7)	
3	3 (10.0)	8 (26.7)	3 (10.0)	

P values represent Chi square test. Given that this was significant for aeroallergens (*), pairwise comparisons were undertaken for aeroallergen sensitization: SPT vs. specific IgE (p = 0.201), specific IgE vs. ISAC (p = 0.023), SPT vs. ISAC (p = 0.119)

More specifically, we compared each participant’s positive sensitizations based on the test performed. We found that 73.3 % of the participants had at least one positive aeroallergen based on their ISAC test compared to both their SPT or sp-IgE results, although the difference was not statistically significant (p = 0.35). For the food allergens, 76.7 and 73.3 % of participants were identified as sensitised by SPT or sp-IgE tests, respectively, compared to their ISAC test but the difference was not significant (p = 0.50).

### Comparison of allergy diagnoses between the two assessors

We then compared allergy diagnoses outcomes between the two different assessors (Table 3). Overall, we found relatively good agreement between the two assessors. There was higher agreement for the food allergens compared to the aeroallergens except for house dust mite and *Alternaria* mould allergy (k = 0.92 and k = 0.70, respectively). The mean number of allergies diagnosed by assessor 1 was 5.3 (SD 2.56) and was 5.9 (SD 2.98) by assessor 2, which were not significantly different (p = 0.20). The distribution of numbers of allergy diagnoses for the two assessors is presented in Table 3. There was no significant difference found between the number of allergy diagnoses for food allergens (p = 0.214) or aeroallergens (p = 0.588) between the two assessors.

**Table 3:** Number (%) of food and aeroallergen diagnoses in 30 participants

Allergy diagnoses	Assessor 1	Assessor 2 (with ISAC results)	Chi square test
*Aeroallergens*			
0	11 (36.7 %)	9 (30.0 %)	0.588
1	4 (13.3 %)	3 (10.0 %)	
2	4 (13.3 %)	4 (13.3 %)	
3	4 (13.3 %)	3 (10.0 %)	
4	5 (16.7 %)	4 (13.3 %)	
5	2 (6.7 %)	2 (6.7 %)	
6	0 (0.0 %)	4 (13.3 %)	
7	0 (0.0 %)	1(3.3 %)	
*Food allergens*			
0	3 (10.0 %)	3 (10.0 %)	0.214
1	7 (23.3 %)	13 (43.3 %)	
2	11 (36.7 %)	4 (13.3 %)	
3	7 (23.3 %)	6 (20.0 %)	
4	2 (6.7 %)	4 (13.3 %)	

As assessment 2 was made with the addition of the ISAC results, we were able to observe the effect the ISAC test had on allergy diagnoses (Table 4). When considering specific allergen diagnoses, a total of 20 diagnoses were removed based on the inclusion of ISAC results and 26 allergen diagnoses were added; 13 of those added were for food allergens not included in the SPT or sp-IgE screens. We found that 70 % (21/30) of the participants had a change in diagnosis between their two assessments with 60 % (18/30) having at least one allergic diagnosis added and 40 % (12/30) having at least one allergic diagnosis removed (Additional file 1: Table S1).

**Table 4:** Allergy assessment by two assessors

Allergic diagnosis	Assessor 1	Assessor 2 (with ISAC results)	Diagnosis removed by assessor 2	Diagnosis added by assessor 2	Agreement between assessors
Atopic dermatitis	30 (100 %)	30 (100 %)	0 (0.0 %)	0 (0.0 %)	1.00
Rhino-conjunctivitis	14 (46.7 %)	8 (26.7 %)	6 (20.0 %)	0 (0.0 %)	0.59 (<0.001)
Asthma	4 (13.3 %)	4 (13.3 %)	0 (0.0 %)	0 (0.0 %)	0.42 (0.010)
Peanut	13 (43.3 %)	13 (43.3 %)	0 (0.0 %)	1 (3.3 %)	0.73 (<0.001)
Egg	15 (50.0 %)	18 (60.0 %)	3 (10.0 %)	1 (3.3 %)	0.67 (<0.001)
Cow’s milk	9 (30.0 %)	7 (23.3 %)	1 (3.3 %)	1 (3.3 %)	0.66 (<0.001)
Other food allergy	21 (70.0 %)	19 (63.3 %)	1 (3.3 %)	13 (43.3 %)	0.85 (<0.001)
Cat	6 (20.0 %)	13 (43.3 %)	3 (10.0 %)	0 (0.0 %)	0.49 (0.001)
Dog	7 (23.3 %)	10 (33.3 %)	1 (3.3 %)	1 (3.3 %)	0.59 (<0.001)
House dust mite	11 (36.7 %)	17 (56.7 %)	2 (6.7 %)	2 (6.7 %)	0.49 (0.001)
*Alternaria* mould	6 (20.0 %)	10 (33.3 %)	1 (3.3 %)	2 (6.7 %)	0.67 (<0.001)
Birch pollen	10 (33.3 %)	13 (43.3 %)	1 (3.3 %)	2 (6.7 %)	0.79 (<0.001)
Timothy grass pollen	13 (43.3 %)	14 (46.7 %)	1 (3.3 %)	2 (6.7 %)	0.53 (0.002)
Horse	1 (3.3 %)	1 (3.3 %)	0 (0.0 %)	1 (3.3 %)	−0.03 (0.575)
Total			14 (46.7 %)	26 (86.7 %)	

Percentages represent the number (%) of participants given each diagnoses by each assessor. The number (%) of participants who had each specific allergy diagnosis removed or added by assessor 2 (impact of the ISAC test results) is also presented. Level of agreement between assessors is presented as Kappa statistics (p values)

With regards to atopy, all the patients had a diagnosis of AD; therefore, there was 100 % agreement between the assessors (k = 1.00). For rhino-conjunctivitis, there were six diagnoses that were removed by assessor 2 and replaced by a diagnosis of non-allergic rhino-conjunctivitis. For asthma, there was no change with regards to diagnosis (k = 0.42) (Table 4).

## Discussion

There is little published data on the use of ISAC testing in AD children. Prosperi et al. [12] found that ISAC testing had reasonable discrimination in detecting allergens associated with asthma, rhino-conjunctivitis, wheeze and airway hypersensitivity but it was not useful for detecting allergens associated with eczema in a paediatric cohort. Our study demonstrates that there is a high prevalence of food and aeroallergen sensitization in children with moderate to severe AD. Although the use of the ISAC-112 testing did not significantly change the number of allergic sensitizations or diagnoses compared to traditional test modalities of SPT and sp-IgE tests, the majority of participants had a change in specific allergy diagnoses when all of the ISAC-112 results were utilized.

Our results demonstrate that for the food allergens tested, sp-IgE testing yielded more positive results as compared to SPT and ISAC, despite the ISAC test covering a greater array of pre-set food allergen components. It has been suggested that the binding characteristics of the ISAC-112 are more specific as they bypass the high rates of sensitization that can occur in AD children who have a raised total IgE. However, the number of food and aeroallergen sensitizations was not lower using ISAC tests, which suggests that the ISAC test may not circumvent this issue.

In recent years, several studies have been performed which demonstrate strong correlation between the ISAC-112 test and other single-plex allergy tests (SPT, sp-IgE tests) [19, 20]. More specifically, studies have shown a good correlation between the testing methods for aeroallergens such as grass pollens [21] and house dust mite in allergic rhinitis patients [11]. In our cohort, the results available from the ISAC-112 test resulted in a change in diagnosis of allergic rhino-conjunctivitis to non-allergic rhino-conjunctivitis for six children. This supports the view that the ISAC test may be helpful at differentiating between allergic and non-allergic rhino-conjunctivitis compared to clinical history and single-plex tests. In fact, the WAO-ARIA-GA^2^LEN consensus guidelines clarify the role that molecular allergy diagnostics such as ISAC-112 can have on resolving genuine versus cross-reactive sensitization in poly-sensitized patients, such as those with AD; hence facilitating a more accurate assessment of the severity of risks to allergens [22].

Another consideration that allergy diagnostic centres may have is the cost of running these diagnostic tests. In our study, SPT cost £15.95 per vial of allergen solution (Alyostal^®^ Stallergenes), which is used for approximately 60 SPT (£0.27 per allergen per test). Skin prick testing requires a trained health care practitioner, safe medical facilities and time to perform and interpret the tests. Sp-IgE tests cost £14.30 per allergen compared to the ISAC test, which costs £250 for 112 allergen components. Both of these tests require phlebotomy services, laboratory staff and the necessary laboratory equipment. For the patients with a clinical history that identifies likely target allergens, confirmation by SPT and sp-IgE testing may prove sufficient and more cost-effective. However, in the poly-sensitised allergic patient with no identifiable causative allergens, based on costing in our tertiary centre, the ISAC test would prove to be more cost effective if the clinician wanted to test for more than 18 allergens or allergen components. Although this seems like a considerable number of allergens to test for, it is not an uncommon practice in investigating AD children. It is in the children with equivocal histories or poly-allergenicity that the ISAC test may provide clinicians with a more comprehensive overview of sensitization to common food and aeroallergens.

## Limitations

The limitations of this study include small sample size recruited in a tertiary allergy clinic, which makes it difficult to extrapolate results to the general population. In smaller hospitals, ISAC testing may not be available which make these results less applicable to general clinical practice. However, our study focused on children with AD, which is an extremely common childhood atopic phenomena, of which allergic diagnoses can be challenging to diagnose. Although we tested for only a small number of allergens, these represent the most common food and aeroallergens in the UK. We assessed the additional diagnostic value of the ISAC-112 as opposed to the diagnostic performance of each of the modalities in isolation against a gold standard diagnostic provocation test. We also acknowledge that it would be beneficial to compare the different test modality outcomes for allergic diagnoses between AD children and a control population (i.e. children without AD) in future work. Also, the fact that the clinical examiners in this study were experienced specialist paediatric allergists can be seen as both an advantage and limitation of the study; this provides greater validity to the clinical diagnoses made, but different allergy tests may be interpreted differently in less experienced hands.

## Conclusions

This pilot study demonstrates that there is a defined role for the use of ISAC testing in diagnosing sensitization and allergy in children with AD, which compares favourably with existing modalities of testing (i.e. SPT, sp-IgE tests). Depending on the clinical setting and the patient population (i.e. those with AD), the ISAC test may be useful in leading to a change in diagnosis and be more cost effective. Further investigation in a larger population, testing each test in isolation as well as against the gold standard (i.e. OFC), is needed.

## Additional file

10.1186/s12948-016-0046-z Diagnoses for individual patients based on inclusion of all ISAC test results.

## Abbreviations

AD: atopic dermatitis
SPT: skin prick tests
Sp-IgE: specific immunoglobulin E
EAACI: European Academy of Allergy and Clinical Immunology
NIAID: National Institute of Allergy and Infectious Diseases
SCORAD: scoring atopic dermatitis system
SD: standard deviation
