# Supplementary material for: Pilot study: assessing the clinical diagnosis of allergy in atopic children using a microarray assay in addition to skin prick testing and serum specific IgE
Source: Clin Mol Allergy. 2016 Aug 19;14:8. doi: 10.1186/s12948-016-0046-z (PMC4991090; doi:10.1186/s12948-016-0046-z)
Supplement: Supplementary file 1 — 10.1186/s12948-016-0046-z Diagnoses for individual patients based on inclusion of all ISAC test results. [file 12948_2016_46_MOESM1_ESM.docx]

**Online Supplemental Table**

**Table S1.** Diagnoses for individual patients based on inclusion of all ISAC test results

| Patient ID | Total Diagnoses Added | Total Diagnoses Removed | Post-ISAC test positive diagnoses |
| --- | --- | --- | --- |
| 1 | 0 | 0 | - |
| 2 | 0 | 0 | - |
| 3 | 0 | 0 | - |
| 4* | 2 | 2 | 0 |
| 5* | 2 | 1 | +1 |
| 6 | 0 | 0 | - |
| 7 | 0 | 0 | - |
| 8 | 0 | 0 | - |
| 9* | 1 | 0 | +1 |
| 10* | 1 | 0 | +1 |
| 11 | 0 | 0 | - |
| 12* | 4 | 0 | +4 |
| 13* | 1 | 5 | -4 |
| 14* | 1 | 0 | +1 |
| 15* | 2 | 0 | +2 |
| 16* | 1 | 0 | +1 |
| 17* | 2 | 1 | +1 |
| 18* | 1 | 0 | +1 |
| 19 | 0 | 0 | - |
| 20* | 0 | 2 | -2 |
| 21* | 1 | 1 | 0 |
| 22* | 0 | 1 | -1 |
| 23 | 0 | 0 | - |
| 24* | 2 | 1 | +1 |
| 25* | 1 | 3 | -2 |
| 26* | 1 | 1 | 0 |
| 27* | 0 | 1 | -1 |
| 28* | 1 | 0 | +1 |
| 29* | 1 | 0 | +1 |
| 30* | 1 | 1 | 0 |
| Total Diagnoses | 26 | 20 |  |

* Participants that had a change in diagnosis based on ISAC results

- = No difference
